# Cancer-driven cytokine immunomodulation ameliorates cardiac function and suppresses fibrosis

**DOI:** 10.1016/j.jmccpl.2025.100493

**Published:** 2025-11-12

**Authors:** Laris Achlaug, Lama Awwad, Irina Langier Goncalves, Sharon Aviram, Ariella Glasner, Ami Aronheim

**Affiliations:** a Department of Cell Biology and Cancer Science, Israel; b Department of Immunology, Israel

**Keywords:** Heart failure, Fibrosis, Innate immunity, Macrophages, Cytokines, Duchenne muscular dystrophy, Cardiac dysfunction

## Abstract

Heart failure remains a leading cause of morbidity and mortality worldwide, with limited progress in the development of novel therapies. It has been demonstrated that tumor growth improves cardiac function and reduces myocardial fibrosis in mouse models of heart failure. It is clear that cancer cell implantation is not a possible therapeutic strategy for heart failure. Therefore, we further studied the underlying mechanism involved, with the objective of demonstrating its broad therapeutic applicability. We show that a single intravenous injection of serum from tumor-bearing mice rapidly augments left-ventricular fractional shortening and suppresses fibrosis in the heart, diaphragm, and skeletal muscles. Cytokine profiling identified IFNγ and TNFα as essential mediators secreted downstream of natural killer (NK) cell activation. Purified recombinant IFNγ and TNFα mimic the serum effect, polarizing cardiac and skeletal macrophages toward an anti-inflammatory, reparative state. We further show that macrophage depletion abrogates the observed beneficial effect, confirming their critical role. Our findings define a novel NK cell–macrophage cytokine axis that reverses cardiac dysfunction and fibrosis in pressure-overload (transverse aortic constriction) and ATF3-transgenic heart failure models. Together, these findings define a novel host-tumor microenvironment response through cytokine secretion, which leads to cardiac repair and dissolution of fibrosis. This work presents a novel therapeutic strategy for harnessing innate immune cells in the treatment of heart failure and fibrotic disease.

## Highlights

• Tumor progression improves cardiac dysfunction and reduces fibrosis.
• Tumor-bearing mouse serum mimics tumor-induced cardioprotection via NK-cell activation.
• NK cell activation promotes IFNγ and TNFα secretion leading to cardiac repair and fibrosis dissolution.
• NK-cells promote macrophage polarization required for tumor-induced cardiac beneficial effects.
• Single injection of purified recombinant IFNγ and TNFα is sufficient to improve cardiac dysfunction and suppress fibrosis.

## Graphical abstract

**Figure:**
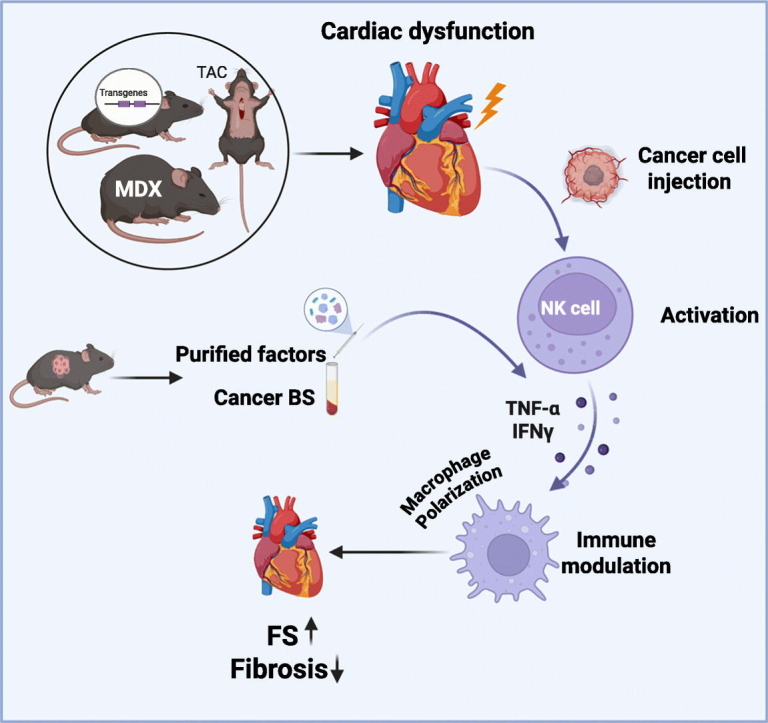
Schematic representation of main findings.

## 1 Introduction

Cardiovascular diseases (CVD) and cancer are the leading causes of death worldwide [[1], [2], [3]]. While CVD and cancer have been considered separate diseases, it is becoming evident that these are highly connected and affect each other's outcomes at multiple levels [4,5]. Cardiac diseases and cancer share similar risk factors, including environmental hazards, genetic predisposition, smoking, obesity, hyperlipidemia, sedentary lifestyle, diabetes, and aging [[6], [7], [8]]. Moreover, these two conditions share pathophysiological mechanisms, such as inflammation and oxidative stress [9]. In recent years, research has focused on the intricate interplay between heart failure and cancer. It is well established that CVD, including heart failure and even early cardiac remodeling, promotes cancer growth and metastatic spread [[10], [11], [12]]. This occurs via multiple mechanisms including cardiac secreted factors [[10], [11], [12], [13]], innate immune alterations [14] and cardiac-derived extracellular vesicles (EVs) [15].

Conversely, recent studies have demonstrated that tumor growth ameliorates heart failure function and suppresses fibrosis, highlighting the bidirectional nature of the interplay between these two pathologies [[16], [17], [18], [19]].

To investigate the impact of tumor growth on cardiac function, the MDX mouse model of Duchenne muscular dystrophy (DMD) was used in combination with cancer cell implantation [20,21]. DMD is a progressive muscle-wasting disorder affecting approximately 1 in 3000 males [22]. The lack of functional dystrophin impairs the interaction and signal transduction between the cytoskeleton and the extracellular matrix (ECM) in skeletal, diaphragm, and cardiac muscles [23]. In a dystrophic heart, cytoskeletal abnormalities can lead to membrane rupture, cardiomyocyte necrosis, and the replacement of contractile myocardium with fibrotic tissue. This process increases wall stress, impairs cardiac function, and leads to heart failure [24]. The MDX mouse model of muscular dystrophy has been widely studied. Although it presents a relatively mild phenotype, it has been proven effective for investigating pathological mechanisms, disease progression, and potential treatments [25]. A previous study showed that tumor-bearing MDX mice exhibited improved cardiac contractile function, as evidenced by increased fractional shortening (FS%) measured by echocardiography [18]. This beneficial outcome was consistent with two additional heart failure mouse models: the activating transcription factor 3 (ATF3) transgenic mouse model for cardiac hypertrophy [17] and transverse aortic constriction (TAC) surgery, a pressure overload mouse model mimicking aortic stenosis [16]. In addition, it was shown that the tumor-mediated beneficial effects are independent of the heart failure-induced tumor promotion [18]. In all of the studies mentioned above, cardiac function was measured by echocardiography performed at a humane endpoint following cancer cell implantation. Here, cardiac contractile function was measured as early as eight days following cancer cell implantation. This allowed the employment of the MDX mouse model for Duchenne muscular dystrophy as a platform to elucidate the molecular mechanisms involved in the tumor-mediated beneficial effects on cardiac dysfunction and suppression of fibrosis [18]. It is shown here that IFNγ and TNFα are both necessary and sufficient to induce the beneficial cardiac effects. These results open a new avenue and potential promise for the treatment of CVDs and fibrotic diseases.

## 2 Materials and methods

### 2.1 Animal protocols

All experimental protocols were approved by the Institutional Committee for Animal Care and Use at the Technion, Israel Institute of Technology, with approval numbers IL-157-10-21, IL-10-02-21, IL-059-04-21, IL-092-07-22. All study procedures comply with the guidelines of the NIH Guide for the Care and Use of Laboratory Animals.

### 2.2 Mouse strains

C57BL/10ScSn-Dmd (MDX), C57BL/10J (B10) and C57Bl/6 (B6) mice were purchased from the Jackson Laboratory. The ATF3 transgenic mouse model is the result of the mating of two transgenic mice: the first expresses the human influenza hemagglutinin (HA) fused to the human ATF3 under the control of the tetracycline activator (tTA) regulatory DNA elements [26]. The second expresses the tTA transcription factor under the control of the αMHC promoter (αMHC-tTA), directing tTA expression to cardiomyocytes [27]. The tTA protein binding to the promoter is regulated by doxycycline (tet-off system). Double transgenic mice containing both the αMHC-tTA and ATF3 transgenes express the human ATF3 in cardiomyocytes and are hereafter designated ATF3 transgenic mice. Hearts, skeletal muscles, diaphragm, and blood were collected for further analysis. Number of mice used in each experiment is indicated in the figure legends. Mice were bred and raised at the Pre-Clinical Research Authority.

### 2.3 Cell culture

The Polyoma Middle T (PyMT) cancer cells were kindly provided by Prof. Tsonwin Hai (Ohio State University, Columbus, OH). PyMT cells are murine breast carcinoma cell lines that were derived from primary tumor-bearing transgenic mice expressing polyoma middle T under the control of the murine mammary tumor virus promoter [28]. Cells were cultured in DMEM containing 10 % FBS, 1 % streptomycin and penicillin, 1 % l-glutamine, and 1 % sodium pyruvate at 37 °C in a humidified atmosphere containing 5 % CO_2_. Cancer cell implantation is used at maximal passage number five.

### 2.4 Cancer cell implantation

PyMT cells (10^6^ cells per mouse) were orthotopically injected into the flanks of male mice. Tumor size was measured using a caliper, and tumor volume was calculated using the formula Width^2^ × Length × 0.5. According to the Institutional Animal Care and Use Committee, the humane endpoint is defined when the maximal tumor size reaches 1500 mm^3^.

### 2.5 TAC surgery

TAC surgery was performed on female and male mice (8–12 weeks old) as previously described [29]. Aortic constriction was achieved using a 27-gauge blunt needle to create a standardized aorta constriction. The TAC operation was performed by an experienced veterinarian who was blinded to the experimental groups. Mice were followed-up until the endpoint (typically three weeks following TAC operation). Only mice that survived the TAC surgery and reached the endpoint were included in the analysis.

### 2.6 Echocardiography

Mice were anesthetized with 1 % isoflurane and kept on a 37 °C heated plate throughout the procedure. Echocardiography was performed with a Vevo3100 micro-ultrasound imaging system (VisualSonics, Fujifilm) equipped with 13- to 38-MHz (MS 400) and 22- to 55-MHz (MS550D) linear array transducers. Cardiac size, shape, and function were analyzed using conventional two-dimensional imaging and M-mode recordings. Maximal left ventricular end-diastolic (LVDd) and end-systolic (LVDs) dimensions were measured in short-axis M-mode images. Fractional shortening (FS) was calculated with the following formula: FS% = [(LVDd − LVDs) / LVIDd] × 100. FS value is based on the average of at least three measurements for each mouse.

### 2.7 RNA extraction

mRNA was extracted from hearts, skeletal muscle, and diaphragms using an Aurum Total RNA fatty or fibrous tissue kit (no. 732-6830, Bio-Rad) according to the manufacturer's instructions. Next, cDNA was synthesized from 1000 ng purified mRNA with the iScript cDNA Synthesis Kit (no. 170-8891, Bio-Rad).

### 2.8 Quantitative real-time PCR

Quantitative real-time polymerase chain (qRT-PCR) was performed with Rotor-Gene 6000 (Bosch Institute, Sydney, Australia) with absolute blue SYBR Green ROX mix (Thermo Scientific AB-4162/B). Serial dilutions of a standard sample were included for each gene to generate a standard curve. Values were normalized to Mb2m expression levels for the heart, diaphragm, and skeletal muscles, and β-actin for the spleen. The sequences of oligonucleotides used are found in Supplementary Table 1.

### 2.9 Fibrosis staining

Heart tissue was fixed in 4 % formaldehyde overnight, embedded in paraffin, serially sectioned at 10-μm intervals, and then mounted on slides. Masson trichrome staining was performed according to the standard protocol. Images were acquired using 3DHistech Pannoramic 250 Flash III (3DHISTECH Ltd). Each section was fully scanned. The percentage of interstitial fibrosis was determined as the ratio of the fibrosis area to the total area of the heart/tumor section using Image Pro Plus software.

### 2.10 Blood serum

Blood was withdrawn from the facial vein with a 4-mm sterile Goldenrod Animal Lancet (MEDIpoint, Inc.). Blood was collected and clotted at room temperature for 2 h, followed by 15 min of centrifugation at 2000*g*. The serum was immediately aliquoted and stored at −20 °C for future use.

### 2.11 Cytokine array

Serum was obtained from either control C57Bl/10J, tumor-bearing C5Bl/10J, cancer-free MDX, or tumor-bearing MDX mice. Serum was applied (75 ml total serum pooled from 5 mice in each group) to probe four Proteome Mouse XL Cytokine Array membranes according to the manufacturer's instruction (R&D systems, Minneapolis, USA; catalog no. ARY028). The signal corresponding to each factor in the array was quantified using TotalLab software analysis. Each protein's expression level on the array was calculated relative to the value obtained for cancer-free C57Bl/10 mice.

### 2.12 Serum inactivation

Serum obtained from mice was heat-inactivated at 56 °C for 15 min. The samples were gently mixed to collect aggregate and centrifuged for 10 min at maximal speed. The supernatant was collected and injected into the tail-vein of MDX mice (100 μl/mouse).

### 2.13 Neutralization of antibodies

Neutralizing antibodies were purchased from Thermo Fisher Scientific: anti-TNFα Monoclonal Antibody (MP6-XT22), anti-IL-6 Monoclonal Antibody (MP5-20F3), anti-GM-CSF Monoclonal Antibody (MP1-22E9), and anti-IFNγ Monoclonal Antibody (XMG1.2). Protein-G PLUS-Agarose beads (sc-2002, Santa Cruz Biotechnology) were used to immune-precipitate the IgG, and the supernatant was injected into the tail-vein of MDX mice. Neutralizing antibodies: IgG antibody (UNLB, 0107-01 SouthernBiotech) was used as control.

### 2.14 Recombinant purified TNFα and IFNγ injection

Purified murine recombinant proteins TNFα (Thermo Fisher cat# 315-01A) and IFNγ (Thermo Cat# 315-05) were added to cancer-free mouse serum to a final 0.5 ng/ml concentration or in saline as indicated. The mixture was injected into the tail-vein of MDX and TAC mice (100 μl). Cancer-free serum was injected into the control MDX and TAC cohort.

### 2.15 CSF1R macrophage depletion

MDX mice were injected twice a week (400 μg per mouse) intraperitoneally (i.p.) with in vivo mAb anti-CSF1R (anti-CD115, BioXCell-BE0213) until the experimental endpoint. Blood was withdrawn after three injections, and at the end of the experiment, spleens were harvested for FACS analysis.

### 2.16 NK cells depletion

MDX mice were injected three times a week with mAb anti-mouse NK1.1 (BioXCell-BE0036) intraperitoneally (i.p.) at 200 μg per mouse injection. Blood was withdrawn at the end of the experiment and spleens were harvested for FACS analysis.

### 2.17 NK cells activation

Mice were injected intraperitoneally (i.p.) with 200 μg PolydIdC (Sigma Aldrich Israel). Blood was withdrawn, spleens were harvested 18 h later, and FACS was used to determine NK cell activation using co-staining with anti-NK.1.1 antibodies (108741, BioLegend) together with either anti-TNFα (560659, BD Pharmingen) or IFNγ (561479, BD Pharmingen).

### 2.18 Intracellular FACS staining of spleen cells

Spleens of mice were harvested and smashed in 10 % DMEM in 6-well plates, after which they were plated in 96-well plates and were incubated with Golgi block solution Brefeldin A and Monensin Solution (1:1000) for 3 h. Subsequently, the plate was centrifuged, washed with PBS (350 ×*g*, 5 min), and stained with the viability dye Ghost Dye-UV 450 (Crytek biosciences 13-0868-T100) at room temperature for 10 min. Stained cells were washed with PBS and centrifuged for 5 min (350 ×*g*). The extracellular stain was applied using anti-NK1.1, anti-CD3, and anti-CD45 antibodies (1:400 in FACS buffer) incubated for 25 min at 4 °C. The cells were washed with PBS by centrifugation and fixed in hotfix/cytoplasm for 15 min at room temperature. Afterward, Intrastain was applied using anti-TNFα-PerCP-Cy5.5 (560659, BD Pharmingen) and anti-IFNγ-APC-Cy7 (561479, BD Pharmingen) antibodies for 30 min at room temperature.

### 2.19 Heart single-cell suspension and flow cytometry

Heart single-cell suspension and flow cytometry were prepared as previously described [30]. Briefly, hearts were perfused, harvested, finely minced, and then incubated with an enzyme mix using the gentleMACS. Cell debris was removed. Afterwards, red blood cells were lysed using ammonium-chloride‑potassium lysis buffer. Next, samples were centrifuged at 400 ×*g* for 5 min at 4 °C, and the pellet was then suspended with FACS buffer. Cells were immune-stained with the following anti-mouse antibodies: CD45-Alexa Fluor 700 (BioLegend, 103128, San Diego, CA, USA), CD11b-PerCP (BioLegend, 101228, CA, USA), F480-PE (BioLegend, 123110, CA, USA), CD206-BV421 (BioLegend, 141717, CA, USA), Ly-6G-Brilliant Violet 510™ (BioLegend, 127633, CA, USA), Ly-6C-PE/Cyanine7 (BioLegend, 128018, CA, USA, CD3-PEcy5 (BioLegend, 100232, CA, USA), NK1.1-BUV395 (BioLegend, 108741, CA, USA), DAPI (Sigma Aldrich 1 μg/μl diluted in PBS) was used for live/dead staining. Cells were incubated (30 min, 4 °C) with the antibody mixture in a staining buffer (PBS containing 1 % bovine serum albumin and 0.05 % sodium azide) and washed twice with a staining buffer. Cells were acquired using an LSRFortessa flow cytometer (BD Biosciences, Franklin Lakes, NJ, USA). The data were analyzed using FlowJo V.10 software (FlowJo, Ashland, OR, USA).

### 2.20 Peripheral blood flow cytometry

Peripheral blood was collected from MDX mice injected with either cancer-derived serum (BS) or control serum and processed for flow cytometry. Blood was anticoagulated with 100 mM EDTA, subjected to red blood cell lysis (1× RBC lysis buffer, 10 min at room temperature), washed with PBS, and resuspended in FACS buffer (PBS with 1 % bovine serum albumin and 0.05 % sodium azide). Cells were stained for 30 min at 4 °C with the following anti-mouse antibodies: CD45-Alexa Fluor 700 (BioLegend, 103128), CD11b-PerCP (BioLegend, 101228), F4/80-PE (BioLegend, 123110), CD206-BV421 (BioLegend, 141717), Ly6G-Brilliant Violet 510™ (BioLegend, 127633), Ly6C-PE/Cyanine7 (BioLegend, 128018), CD3-PE/Cy5 (BioLegend, 100232), and NK1.1-BUV395 (BioLegend, 108741). After two washes, cells were acquired using an LSRFortessa flow cytometer (BD Biosciences, Franklin Lakes, NJ, USA), and data were analyzed with FlowJo v10 (FlowJo, Ashland, OR, USA).

### 2.21 Statistical analysis

Data are presented as mean ± SE. All mice were included in each statistical analysis unless they were euthanized for humane reasons before the experimental endpoint. Experimental groups were blinded to the experimentalists during data collection. Animals were selected for each group in a randomized fashion. Number of mice in each experimental group is at least *n* = 3. The statistical significance of tumor volume was determined by two-way repeated-measures ANOVA followed by the Bonferroni posttest. Comparison between several means was analyzed by one-way ANOVA followed by the Tukey posttest. Comparison between two means was performed by two-tailed Student *t-*test. Analyses were performed with GraphPad Prism 10 software. Values of *p* < 0.05 were accepted as statistically significant.

## 3 Results

### 3.1 Serum from tumor-bearing mice mimics the cancer cells' cardiac beneficial effects

To investigate the effect of tumor growth on cardiac dysfunction in the MDX mouse model, we implanted PyMT breast cancer cells in five-month-old mice (Supplemental Fig. 1A). Cardiac function was assessed via echocardiography as early as eight days post-injection. Tumor volume was followed over the experimental timeline (Supplemental Fig. 1A). Interestingly, an improvement in cardiac contractility, as reflected by increased fractional shortening (FS %), was observed as early as eight days following cell implantation, even before a measurable tumor developed (Supplemental Fig. 1A–B, Supplemental Table 2). This suggests a rapid systemic effect of cancer cells on cardiac function, independent of tumor size. Based on this, we hypothesized that a soluble factor might be responsible for the observed beneficial cardiac effect.

**Fig. 1:**
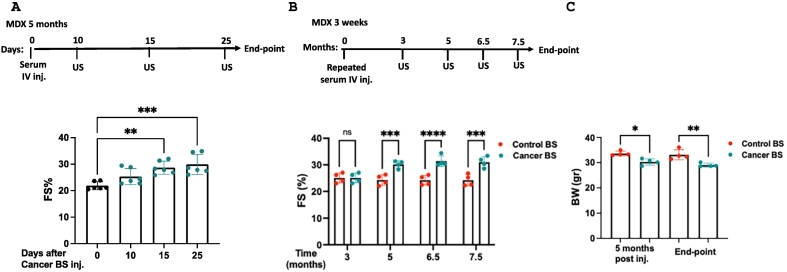
Serum derived from tumor-bearing MDX mice ameliorates cardiac function. (A) Schematic representation of the experimental timeline. MDX male mice (5 months old, n = 6) were tail-vein injected (IV inj.) with 100 μL each with blood serum (BS) derived from tumor-bearing MDX mice (Cancer BS). Echocardiography (US) was performed pre-injection (0 days), 10, 15, and 25 days post-serum injection, and fractional shortening (FS) was calculated. (B) Three-week-old mice (n = 4/group) were injected with either control blood serum (Control BS) or Cancer BS repeatedly every 30 days from three weeks of age until the endpoint (eight months of age). Echocardiography (US) was performed, and FS was calculated at the indicated time points (C). Body weight (BW, gr) measurement of MDX mice (n = 4/group) at 5 months post-injection and at the end-point. Results are presented as mean ± SEM; one-way repeated measures ANOVA followed by (A, C) Tukey posttests or (B) two-way ANOVA followed by Bonferroni post-test. *p < 0.05; **p < 0.01; ***p < 0.001; ****p < 0.0001. Each dot represents one mouse.

To test this, we administered a single intravenous (IV) injection of serum (100 μl) derived from tumor-bearing mice into five-month-old MDX mice. Remarkably, cardiac contractile function improved within 15 days and was sustained for at least 25 days (Fig. 1A, Supplemental Table 3). This improvement was accompanied by reduced expression of hallmark fibrosis gene markers in the heart and diaphragm muscles compared to serum from naïve MDX mice (Supplemental Fig. 2A–B). In addition, we observe a systemic anti-inflammatory polarization of macrophages (Supplemental Fig. 2C). To evaluate the long-term effects, we initiated repeated serum injections every 30 days from weaning (three weeks old) until eight months of age. Mice receiving serum derived from tumor-bearing mice exhibited sustained improvements in cardiac contractility compared to controls, along with reduced body weight, likely due to increased cage activity (Fig. 1B–C, Supplemental Table 4).

**Fig. 2:**
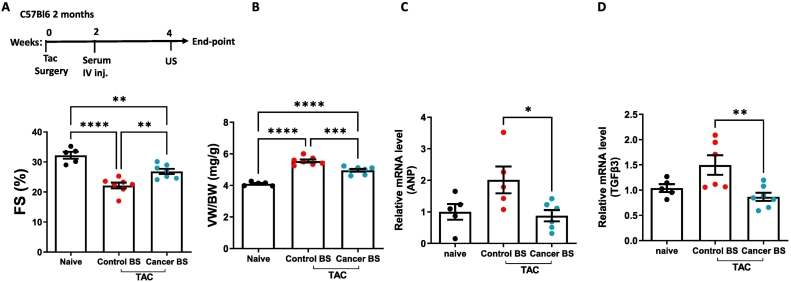
Tumor-bearing mice's blood serum ameliorates cardiac dysfunction following TAC surgery. (A) Experimental timeline. Naïve (n = 5) or TAC-operated two-month-old C57Bl/6 mice were injected with either Control BS (n = 7) or Cancer BS (n = 7). Echocardiography was performed prior to sacrifice, and Fractional shortening (FS%) was calculated. (B) Ventricles to body weight ratio (mg/g) at endpoint. (C–D) Levels of hypertrophy (ANP) and fibrosis (TGFβ3) hallmark genes were measured in qRT-PCR using cDNA derived from heart mRNA of each mouse. Results were normalized to Hsp90 housekeeping gene and compared to naïve levels (non-operated mice). Results are presented as mean ± SEM, One-way ANOVA followed by Tukey post-test was performed. *p < 0.05, **p < 0.01, ***p < 0.001, ****p < 0.0001. Each dot represents one mouse.

Previously, similar tumor-induced cardiac beneficial effects in two other heart failure models: transverse aortic constriction (TAC) [16] and the ATF3 transgenic mouse model [17]. To determine whether cancer serum, regardless of the solid tumor, can replicate these effects, we injected serum derived from tumor-bearing mice two weeks post-TAC surgery. We analyzed cardiac function at the study endpoint (Fig. 2A, Supplemental Table 5). Consistently, mice receiving serum derived from tumor-bearing mice exhibited improved cardiac function, as indicated by increased FS% (Fig. 2A, Supplemental Table 5) and reduced ventricle weight-to-body weight ratio (Fig. 2B). Additionally, suppression of cardiac hypertrophy was demonstrated by decreased expression of the hypertrophic marker ANP (Fig. 2C) and the fibrosis-associated gene TGFβ3 (Fig. 2D). Similar cardiac contractile function improvements were observed in ATF3 transgenic mice following injections of serum derived from tumor-bearing mice (Supplemental Fig. 3, Supplemental Table 6). In contrast, serum derived from tumor-free mice showed no effect (Supplemental Fig. 3, Supplemental Table 6).

These findings suggest that serum derived from tumor-bearing mice mimics the effects of cancer cell implantation, enhancing cardiac contractile function, reducing hypertrophy, and accelerating the resolution of fibrosis.

### 3.2 NK cell activation mediates cancer cell growth

**Fig. 3:**
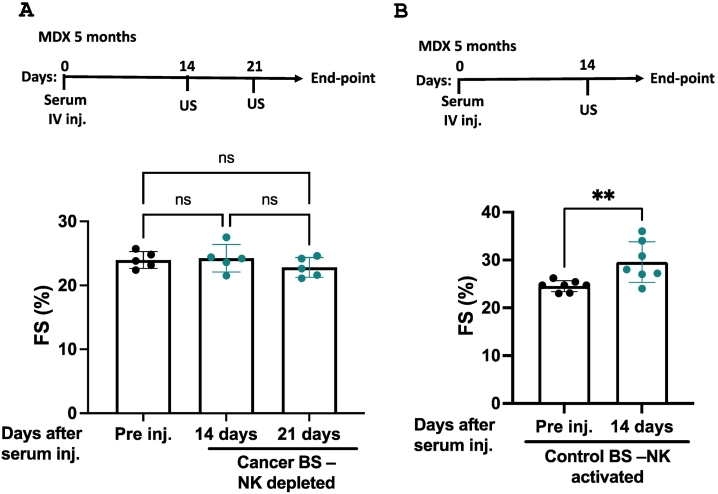
NK cells are necessary and sufficient for ameliorating the cardiac contractile function of MDX mice. (A) Experimental timeline. Five-month-old MDX mice (n = 5) were tail-vein-injected with serum derived from NK-depleted tumor-bearing mice. Echocardiography was performed pre- and post-injection (14 and 21 days). Fractional shortening (FS%) was calculated. (B) Experimental timeline. C57Bl/6 were injected with Poly dIdC, and serum was withdrawn 18 h later. Subsequently, five-month-old MDX mice (n = 7) were tail-vein-injected with serum derived from Poly dIdC injected mice. Results are presented as mean ± SEM; one-way repeated measures ANOVA followed by (A) Tukey posttests or (B) Student's *t*-test. **p < 0.01. (ns) No statistical significance. Each dot represents one mouse.

To further investigate the molecular mechanisms underlying this beneficial effect, we analyzed the serum composition. Blood serum contains various soluble components, including proteins, exosomes, extracellular vesicles, metabolites, and miRNAs [31]. We performed selective exosome depletion experiments to determine whether exosomes or proteins play a key role in mediating this phenotype. Exosome depletion did not impact serum-induced cardiac beneficial effects (Supplemental Fig. 4A, Supplemental Table 7). In contrast, heat inactivation, where proteins were denatured, abolished these effects (Supplemental Fig. 4B, Supplemental Table 8), suggesting that proteins mediate the tumor-dependent cardiac effect. To test whether cancer cell-secreted proteins alone were sufficient, we injected MDX mice with conditioned medium (CM) collected from PyMT breast cancer cultured cells. However, CM had no significant effect on cardiac contractility (Supplemental Fig. 4C, Supplemental Table 9). Further, serum was collected at different time points post-cancer cell implantation. Serum derived from mice two or 30 days post-cancer cell implantation had no effect. In contrast, serum derived following six days post-implantation demonstrated a beneficial effect (Supplemental Fig. 4D, Supplemental Table 10). These results suggest that the beneficial effect stems from a host-derived humoral response rather than direct cancer cell secretion. Given that the adaptive immune system was previously ruled out as a key mediator [16], we focused on innate immune components such as natural killer (NK) cells. NK cells, known for their early tumor-suppressive activity, were investigated for their potential involvement. Thus, NK cells were depleted using anti-NK1.1 antibodies before tumor implantation. Serum was collected 14 days later, and flow cytometry confirmed NK cell depletion in the blood and spleen (Supplemental Fig. 5A–B). Serum derived from tumor-bearing NK cell-depleted mice was injected into five-month-old MDX mice (Fig. 3A, Supplemental Table 11). The NK cell-depleted serum failed to improve cardiac function, suggesting that NK cells are essential for the humoral response to cancer cell implantation. Conversely, NK cells were activated by the injection of Poly dIdC. Serum collected from these mice mimicked the beneficial cardiac effects equally well compared to serum derived from tumor-bearing mice (Fig. 3B, Supplemental Table 12).

### 3.3 INFγ and TNFα are necessary and sufficient for the amelioration of cardiac dysfunction

**Fig. 4:**
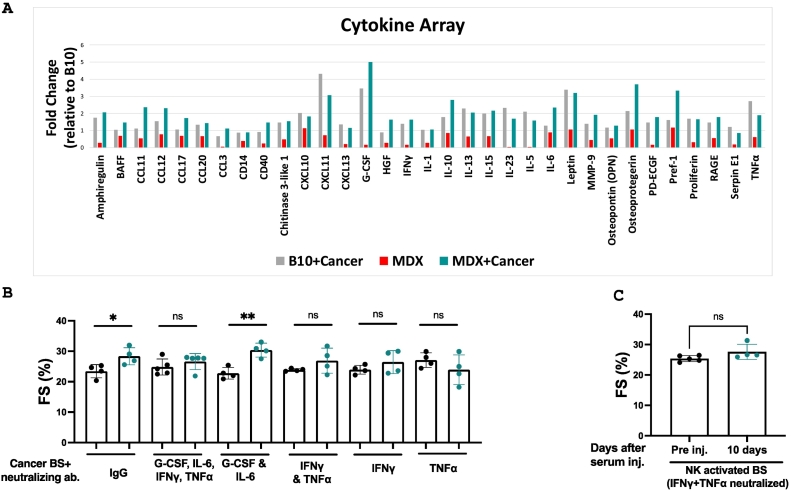
IFNγ and TNFα are responsible for ameliorating cardiac contractile function in MDX mice. (A) Cytokine array proteome profiler membranes were incubated with serum (pooled from 5 mice/cohort) derived from C57Bl/10 control mice (B10) and MDX mice in the presence or absence of a tumor. Serum was extracted 14 days following cancer cell implantation. The expression level of each cytokine present in C57Bl/10 mice was considered 100 %, and all other groups are presented relative to this. (B) Serum derived from tumor-bearing mice was incubated with IgG-neutralizing antibodies against the indicated candidate factors or with an equivalent amount of IgG control. Subsequently, serum was injected into the tail vein of five-month-old MDX mice (n = 4–5/group). Echocardiography was performed pre- (black dots) and 10 days post-serum injection (turquoise dots). Fractional shortening (FS%) was calculated. (C) Serum derived from Poly dIdC-injected mice was incubated with neutralizing antibodies against IFNγ and TNFα. Serum was injected into the tail vein of five-month-old MDX mice (n = 5). Echocardiography was performed pre- and 10 days post-serum injection. Fractional shortening (FS%) was calculated. Results are presented as mean ± SEM *p < 0.05, **p < 0.01. *p* > 0.05. No statistical significance (ns). Each dot represents one mouse. (For interpretation of the references to color in this figure legend, the reader is referred to the web version of this article.)

To identify humoral factors responsible for these effects, we performed a proteome profiler cytokine array using serum from C57Bl10 (MDX genetic background) and MDX mice with and without cancer (Fig. 4A, Supplemental Fig. 6). We prioritized cytokines at significantly higher levels in serum derived from tumor-bearing mice compared to tumor-free MDX mice. Subsequently, we tested candidate cytokines by using specific neutralizing antibodies to block cytokine action in the serum. We first tested four pro-inflammatory cytokines IL-6, G-CSF, TNFα, and IFNγ as potential candidates. Cancer serum was incubated with control IgG or neutralizing antibodies targeting IL-6, G-CSF, TNFα, and IFNγ before injection into MDX mice. Depletion of TNFα and IFNγ, but not IL-6 or G-CSF, abolished the beneficial cardiac effects (Fig. 4B, Supplemental Table 13). We used flow cytometry to determine whether these cytokines are also elevated in serum activated by NK cells. This analysis confirmed increased TNFα and IFNγ levels in NK cells from Poly dIdC-treated mice compared with non-injected control mice (Supplemental Fig. 7). Indeed, the neutralization of these cytokines in NK cell-activated serum completely abrogated the cardiac beneficial effect (Fig. 4C, Supplemental Table 14).

**Fig. 5:**
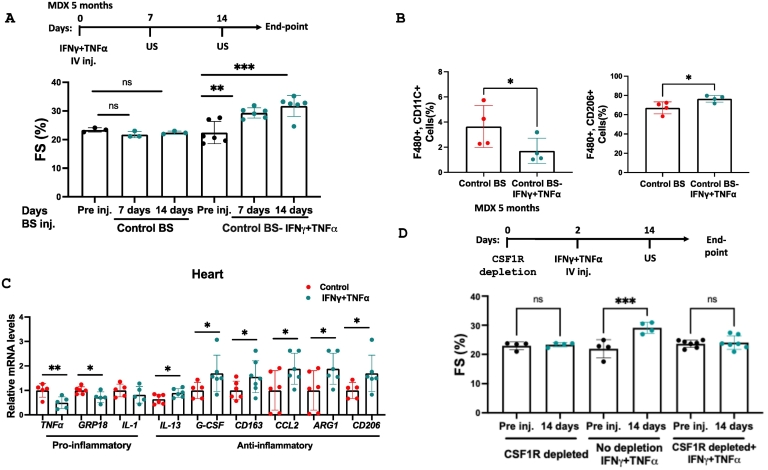
Purified IFNγ and TNFα improve cardiac contractile function via macrophages polarization in MDX mice in a CSF1R macrophages-dependent manner. (A). Experimental timeline. Five-month-old MDX mice were tail-vein-injected with serum either Control BS (n = 3) or Control BS supplemented with purified IFNγ and TNFα (n = 6). Echocardiography was performed pre- and post-injection (7 and 14 days). Fractional shortening (FS%) was calculated. (B) Flow cytometry analysis quantifying cardiac macrophages (% F4/80^+^CD11c^+^ and F4/80^+^CD206^+^ respectively) in control (n = 4) and IFNγ + TNFα-injected (n = 4) MDX mice. (C) qRT-PCR measuring transcription levels of macrophage hallmark gene markers in the heart of MDX mice injected with serum supplemented with IFNγ and TNFα compared with naïve mice serum (n = 5–7/group). Data are presented as the relative expression compared to MDX mice injected with serum only (determined as a mean of 1). (D) Experimental timeline. Five-month-old MDX control (no depletion, n = 4) and CSF1R macrophages-depleted mice (n = 7) were tail vein injected with control BS supplemented with IFNγ and TNFα. CSF1R-depleted mice (n = 4) were left untreated. Echocardiography was performed pre- (pre inj.) and post-cytokine injection (14 days). Fractional shortening (FS%) was calculated. Data are presented as mean ± SEM. One-way repeated measures ANOVA followed by Tukey posttests (C), Student's *t*-test (B), or two-way ANOVA with Bonferroni repeated measures (A, D). *p < 0.05, **p < 0.01, ***p < 0.001. *p* > 0.05 no statistical significance (ns). Each dot represents one mouse.

To assess whether TNFα and IFNγ alone were sufficient to induce cardiac improvement, we injected purified recombinant murine TNFα and IFNγ into five-month-old MDX mice. This treatment replicated the effects of tumor-bearing serum, improving cardiac function (Fig. 5A, Supplemental Table 15), and reducing the transcription of hallmark gene markers of fibrosis in the heart, diaphragm, and skeletal muscles in MDX mice (Supplemental Fig. 8A–C). In addition, a significant reduction in fibrosis following Masson trichrome staining of heart sections (Supplemental Fig. 8D).

### 3.4 INFγ and TNFα mediate macrophage polarization, which is necessary for cardiac repair and regeneration

Previous studies suggested that cancer cell implantation promotes anti-inflammatory macrophage polarization [16,18]. Indeed, flow cytometry analysis revealed a reduction in pro-inflammatory macrophages and an increase in anti-inflammatory macrophages in the heart following TNFα and IFNγ injection (Fig. 5B, Supplemental Fig. 9). This was accompanied by qRT-PCR analysis of multiple inflammatory hallmark gene markers (Fig. 5C). To evaluate the acute effects of INFγ and TNFα injection, we measured the expression of hallmark pro-inflammatory and immediate-early stress response gene (ATF3) in the heart and spleen 24 h post-treatment. Strikingly, most pro-inflammatory transcripts were suppressed, and the stress-responsive gene ATF3 remained uninduced (Supplemental Fig. 10(A–B). The low level of expression of the pro-inflammatory genes was sustained for two weeks as well (Supplemental Fig. 10C).

To study the potential signaling pathways that are changed following INFγ and TNFα treatment, we examined the expression level of TNFR 1&2 and their target genes by qRT-PCR. We observed a shift in the balance of TNF 1&2 receptor signaling two weeks following INFγ and TNFα injection. Specifically, TNFR1 (Tnfrsf1a) expression is significantly reduced, along with downstream pro-inflammatory markers such as TNFα, CD163, and IL-1β, indicating suppression of the canonical inflammatory TNFR1 axis. In contrast, TNFR2 (Tnfrsf1b) expression is maintained or upregulated, suggesting a bias toward the reparative, pro-survival TNFR2 pathway (Supplemental Fig. 11). Importantly, the stress-inducible gene ATF3 is not induced, further supporting the conclusion that this cytokine treatment dampens immediate stress and inflammatory responses while redirecting TNF signaling toward a reparative phenotype.

**Fig. 6:**
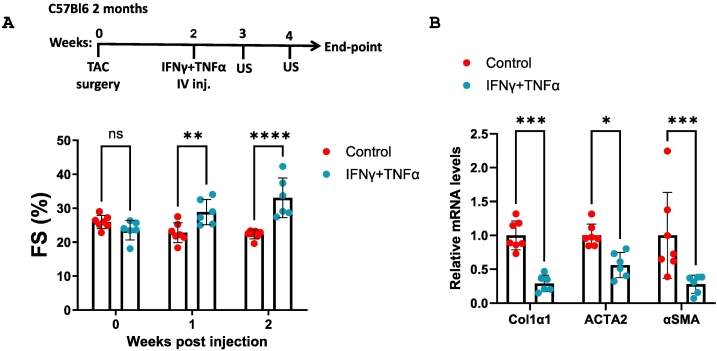
IFN-γ and TNF-α administration enhance cardiac function and suppress fibrosis following TAC surgery. (A) Experimental timeline. TAC-operated two-month-old C57Bl/6 mice were injected with either Control BS (n = 7) or Cancer BS supplemented with IFN-γ and TNF-α (n = 6). Echocardiography was performed prior to, one, and two weeks post-injection, and fractional shortening (FS%) was calculated. (B) Levels of fibrosis hallmark genes Col1α1, ACTA2 and αSMA were measured in qRT-PCR using cDNA derived from the heart mRNA of each mouse. Results were normalized to Hsp90 housekeeping gene. Results are presented as mean ± SEM, One-way ANOVA followed by Tukey post-test *p < 0.05, **p < 0.01, ***p < 0.001, ****p < 0.0001. *p* > 0.05 no statistical significance (ns). Each dot represents one mouse.

To determine whether macrophages act downstream of INFγ and TNFα in mediating beneficial cardiac effects, we depleted CSF1R-expressing macrophages in MDX mice using repeated anti-CD115 antibody injections. Strikingly, macrophage depletion abolished the contractile improvements induced by TNFα and IFNγ, demonstrating that macrophages are essential downstream mediators of cytokine-driven cardiac repair (Fig. 5D, Supplemental Table 16). Similarly, we used purified recombinant IFNγ and TNFα injections in mice two weeks following TAC surgery (Fig. 6A). These improved contractile functions and reduced hallmark fibrosis gene markers (Fig. 6A–B, Supplemental Table 17). Collectively, purified recombinant IFNγ and TNFα could fully recapitulate the effects of tumor-bearing serum.

## 4 Discussion

Heart failure and fibrotic diseases remain significant clinical challenges and major public health concerns. Despite substantial medical advancements, no novel therapeutic strategies have emerged in the past decade [32]. Recent studies have highlighted the complex interplay between cardiovascular diseases and cancer [19]. These studies demonstrated improved cardiac function in tumor-bearing mice [[16], [17], [18]]. In the current study, the underlying mechanisms of this phenomenon were elucidated.

Our findings reveal that tumors confer a rapid, beneficial cardiac effect, which can be replicated through serum administration derived from tumor-bearing mice. Repeated injections of cancer-derived serum preserved the cardiac dysfunction amelioration in a preventive approach for long-term treatment. This enhanced cardiac pathophysiology and reduced fibrosis in skeletal and diaphragm muscles. MDX mice tend to weigh more than healthy mice, primarily due to increased muscle fibrosis and decreased physical activity [33]. Interestingly, these treatments significantly reduced the mice's weight. The observed reduction in body weight following serum treatment may reflect an overall improvement in muscle function, potentially linked to reduced fibrotic burden and enhanced mobility.

To uncover the mechanism underlying the tumor-induced cardiac phenotype, we identified a novel interaction between tumor and cardiac function. Heart failure has been shown to promote tumor growth through multiple mechanisms including: secreted factors [[10], [11], [12], [13]], immune cell modulation [14], and extracellular vesicles [15]. Our results demonstrated that exosomes are not involved in mediating the tumor-dependent cardiac-enhancement phenotype. Instead, we showed that tumor-induced secreted cytokines play a crucial role in modulating the innate immune system.

Our study shows that cancer cells are initially recognized by NK cells. This temporal dynamic is critical, as serum from tumor-bearing mice lacking NK cells failed to improve MDX cardiac function, underscoring the essential role of NK cells. Notably, serum derived from NK cell-activated mice using Poly dIdC was sufficient to recapitulate these cardioprotective effects. This approach bypassed the need for cancer cell implantation. Further analysis identified the CSF1R macrophage population as key mediators of this reparative effect.

Additionally, we found that serum derived from tumor-bearing mice for 30 days failed to confer beneficial cardiac effects. This time-dependent limitation may explain why previous heart failure models, such as long-term myocardial infarction studies, failed to observe similar cardioprotective effects by growing tumors [13,14]. In human cancer patients, early treatment upon diagnosis may obscure potential beneficial cardiac outcomes due to immune modulation. Moreover, most cancer therapies are associated with cardiotoxicity [34], potentially masking any innate cardioprotective effects observed in cancer patients.

Our findings reveal a novel NK cell-macrophage axis in cardioprotection. This axis is driven by secreted cytokines, with IFNγ and TNFα, playing a pivotal role in promoting a regenerative phenotype and reduced fibrosis. These cytokines induce cardiac macrophage polarization toward an anti-inflammatory state, thereby facilitating cardiac repair and resolution of fibrosis. This is consistent with a previous study on skeletal muscle repair in MDX mouse model of Duchenne muscular dystrophy [35]. TNFα is a key inflammatory cytokine elevated in many CVDs. It signals through two receptors: TNFR1 (pro-inflammatory and pro-apoptotic) and TNFR2 (regenerative and immune modulating) [36]. This suggests that excess administration of TNFα tilts the balance toward TNFR2 which associates with the regenerative and repair program.

Similarly, IFNγ is a pro-inflammatory cytokine recognized as a central regulator of immune responses. It exerts broad effects on T cells, NK cells, and macrophages, enhancing their antiviral and antimicrobial functions and promoting the expression of major histocompatibility complex (MHC) class I molecules on macrophages [37]. Our findings suggest that a single injection of TNFα and IFNγ induces immune cell polarization toward an anti-inflammatory state in the cardiac tissue that drives tissue repair.

The MDX mouse model played a crucial role in identifying the key components responsible for these beneficial cardiac effects. However, it is essential to acknowledge its limitations. While the MDX model is valuable for studying cardiac, diaphragm, and skeletal muscle fibrosis, it does not fully replicate the cardiac hypertrophy typically associated with heart failure [21]. Nonetheless, our findings were recapitulated in two other heart failure models, increasing the generality and relevance of our results. We propose a novel therapeutic approach to mitigate fibrosis and enhance cardiac function by activating NK cells and promoting an immunomodulatory phenotype that fosters repair. The co-administration of IFNγ and TNFα is central to these cardio-protective effects, ensuring systemic beneficial effects. We examined the effects of IFNγ and TNFα treatment on the expression of inflammatory and stress-response genes in the heart and spleen at 24 h and 14 days post-administration. While these analyses provide initial insights, a more comprehensive tissue evaluation will be required to understand the potential side effects of prolonged cytokine-based treatments and optimize their therapeutic application. It should be noted, however, that chronic administration of proinflammatory cytokines such as IFNγ and TNFα may have harmful, unwanted side effects. In contrast, we used a single injection of purified cytokines with a very short half-life [38,39], thereby minimizing the risks of safety and toxicity.

## 5 Conclusions

Our study identifies a novel immunomodulatory mechanism by which tumors confer cardioprotection through NK cell activation and macrophage polarization. Tumor-secreted cytokines, IFNγ and TNFα, drive cardiac repair and suppress fibrosis. These findings highlight potential translation opportunities. Further evaluation of cytokine-based interventions for safety and long-term efficacy is needed.

### 5.1 Limitations

Although the MDX mouse model was essential in delineating the mechanisms underlying the cardioprotective effects of tumor-derived factors, it only partially reflects the full spectrum of human Duchenne cardiomyopathy and does not reproduce the hypertrophic phenotype observed in many forms of human heart failure. To mitigate this, we validated our findings in two additional models (TAC and ATF3-transgenic mouse models); nevertheless, translation to human disease remains to be established. In addition, safety considerations, including the potential long-term safety of sustained cytokine exposure, even though we used a single injection of short-lived cytokines. This was not addressed in the current study. These issues warrant further investigation in future work, particularly in the context of translation to human therapeutic applications.

## CRediT authorship contribution statement

**Laris Achlaug:** Writing – review & editing, Methodology, Investigation, Formal analysis, Data curation, Conceptualization. **Lama Awwad:** Writing – review & editing, Methodology, Investigation, Formal analysis, Data curation, Conceptualization. **Irina Langier Goncalves:** Methodology, Investigation. **Sharon Aviram:** Resources, Methodology. **Ariella Glasner:** Investigation, Conceptualization. **Ami Aronheim:** Writing – original draft, Visualization, Validation, Supervision, Resources, Project administration, Investigation, Funding acquisition, Conceptualization.

## Declaration of Generative AI and AI-assisted technologies in the writing process

The authors did not use generative AI or AI-assisted technologies in the development of this manuscript.

## Declaration of competing interest

The authors declare no competing interests.

## Data Availability

The data generated in this study are available upon request from the corresponding author.

## Appendix A Supplementary data

Supplementary figures Image 1

Supplementary tables Image 2
