# Supplementary material for: Cancer-driven cytokine immunomodulation ameliorates cardiac function and suppresses fibrosis
Source: J Mol Cell Cardiol Plus. 2025 Nov 12;14:100493. doi: 10.1016/j.jmccpl.2025.100493 (PMC12664467; doi:10.1016/j.jmccpl.2025.100493)
Supplement: Supplementary file 1 — Supplementary figures [file mmc1.docx]

**Supplemental Figures and Legends**

**
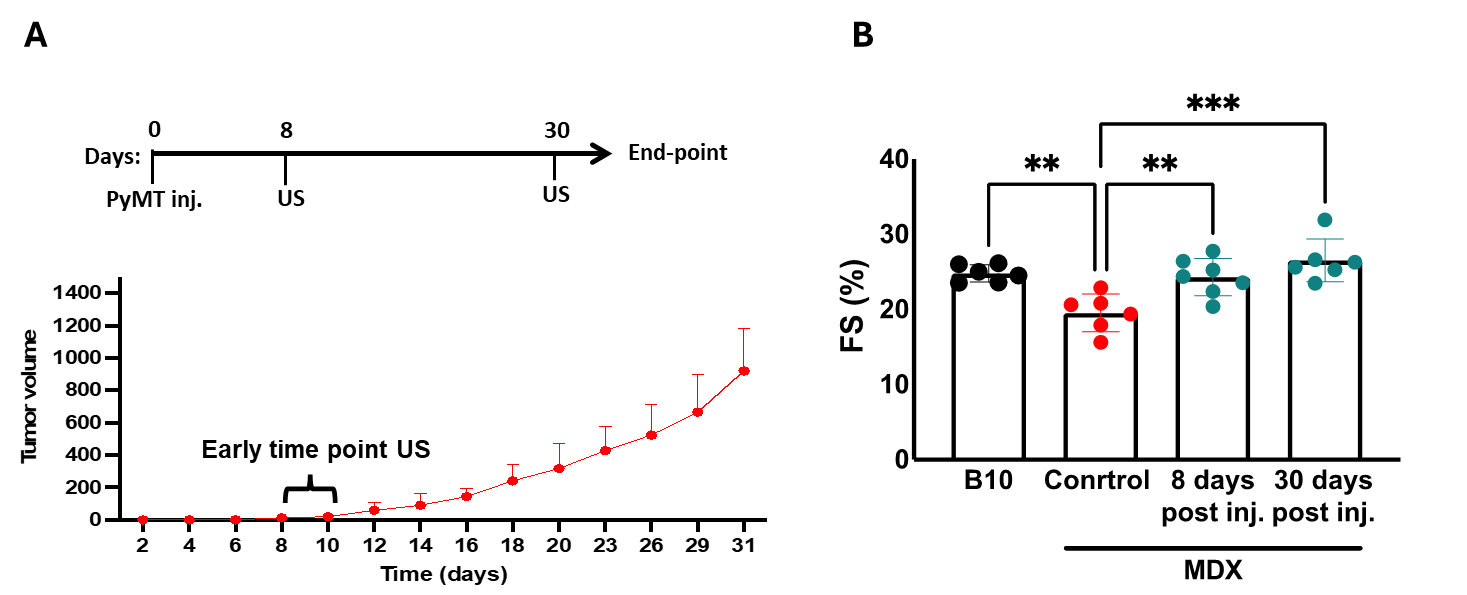
**

**Supplemental Figure 1. PyMT breast cancer cells injection improves heart function in MDX male MDX mice 8 days post-injection.** **(A)** Experimental timeline. Five-month-old male mice (n=6-7/group) were injected in the flanks with PyMT cells (10^6^ cells per mouse) or left untreated (control, n=6). Tumor volume was monitored (width^2^ X length X 0.5) over time in the MDX mice. **(B)** Echocardiography was performed 8 days and 30 days post-injection. Fractional shortening (FS) was calculated in C57Bl/10 (B10), tumor-free MDX (MDX), and tumor-bearing MDX mice. Results are presented as mean ± SEM one-way repeated measures ANOVA followed by Tukey. *** p < 0.01; *** p < 0.001*. Each dot represents one mouse.


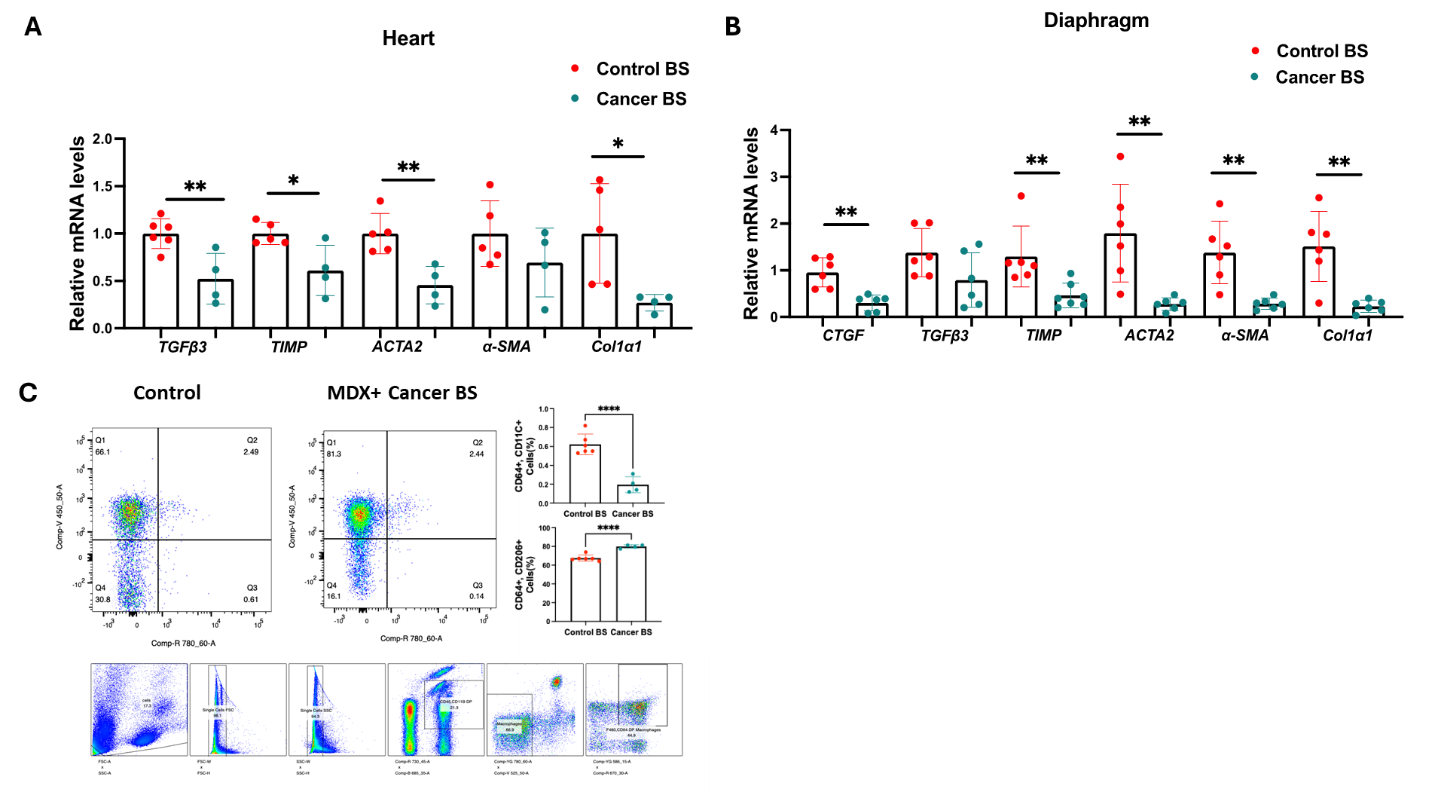


**Supplemental Figure 2**. **Serum derived from tumor-bearing mice induces macrophage polarization, resulting in fibrosis suppression in the heart and diaphragm muscles**. MDX male mice (5 months old) were tail-vein injected (IV inj.) with 100 µL each with either control blood serum (Control BS, n=5-6) or serum derived from tumor-bearing MDX mice (Cancer BS, n=4). **(A, B)** The transcription levels of fibrosis hallmark gene markers in the **(A)** heart and **(B)** diaphragm were measured using qRT-PCR normalized to mb2m. Data is shown as the relative expression compared to MDX control mice (determined as 1). **(C)** Flow cytometric analysis of peripheral blood from MDX mice injected with cancer-derived serum (n = 4) or control blood serum (n = 4), quantifying macrophages (% CD64⁺CD11c⁺ and CD64⁺CD206⁺ , respectively). Results are presented as mean ± SEM; One-way repeated measures ANOVA followed by Tukey (A-B) or Student’s t-test (C). * *p* < 0.05; ** *p* < 0.01. Each dot represents one mouse.


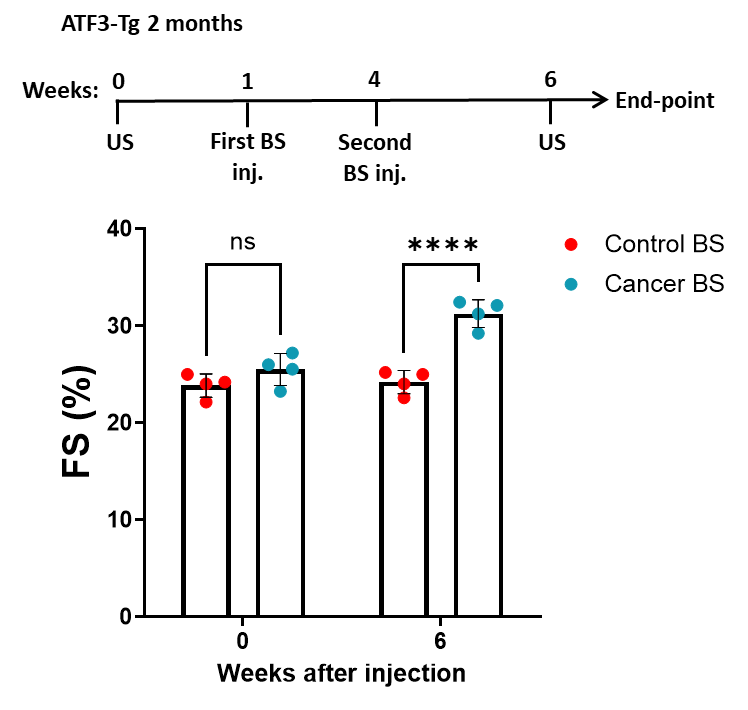


**Supplementary Figure 3. Tumor-bearing mice's blood serum enhances cardiac contractility function in the ATF3 transgenic mouse model.**  Experimental timeline. Two-month-old ATF3 transgenic mice were injected twice with a three-week interval with either control BS or Cancer BS (n=4/group). Echocardiography was performed prior to injection and six weeks post-injection. Fractional shortening (FS%) was calculated.
Results are presented as mean ± SEM, One-way ANOVA followed by Tukey posttest. ****P<0.0001, p>0.05 No statistical significance (ns). Each dot represents one mouse.

**Supplemental Figure 4. Secreted factors are responsible for ameliorating cardiac contractile function in MDX mice.** Echocardiography (US) was performed pre-injection and 10 days post-serum injections in each experiment. **(A)** MDX mice were injected with a serum derived from cancer-free (n=3) and a serum derived from tumor-bearing mice-deprived exosomes following differential centrifugation (n=5). **(B)** MDX mice (n=4) were tail vein injected with either control serum (cancer-free) derived from C57/Bl/10 mice, tumor-bearing C57/Bl/10 mice (cancer), or heat-inactivated serum derived from tumor-bearing C57/Bl/10 mice (cancer heat-inactivated). **(C)** MDX mice (n=3) were injected with conditioned medium (CM) collected from PyMT breast cancer cultured cells**. (D)** Serum derived from cancer cells implanted in mice was collected after two, six and 30 days. Serum was injected into the tail vein of MDX mice (n=3-4/group), and FS% was calculated. Data are presented as mean ± SEM. one-way repeated measures ANOVA followed by **(A, B, D)** Tukey posttests or **(C)** Student’s t-test. **p<0.05 ** p < 0.01, ****p<0. 0001. p>0.05* no statistical significance (ns). Each dot represents one mouse.

**Supplemental Figure 5. NK cell depletion in the spleen and blood of MDX mice.** Mice were injected three times a week with anti-NK1.1 for four weeks. Subsequently, PyMT cells were implanted, and blood was withdrawn 14 days later. **(A)** NK depletion was evaluated by FACS analysis in the blood after three injections. At the humane endpoint, NK cell depletion was evaluated by FACS in the **(B)** blood and **(C)** spleen of either control (n=3-5/group) or NK-depleted MDX mice (n=4-5/group). The number of NK1.1 cells in the control was determined as 1. Results are presented as mean ± SEM; Student’s t-test. ** p < 0.05; ** p < 0.01.* Each dot represents one mouse.

**Supplemental Figure 6**. **Complete data of Cytokine array proteome profiler**. **(A, B)** Membranes were incubated with serum (pooled from 5 mice/cohort) derived from C57/Bl/10 control mice, tumor-bearing C57/Bl/10 mice, tumor-free MDX, and tumor-bearing MDX mice. The expression level of each cytokine present in C57Bl/10 mice was considered 100% and all other groups are presented relatively.

**Supplemental Figure 7. IFNγ and TNFα expression in NK cells derived from Poly dIdC-injected mice.** Mice were injected with Poly dIdC, and 18 hours later, blood was withdrawn. NK cell activation was assessed by FACS using anti-NK1.1 together with either anti-INFγ or anti-TNFα antibodies. The percentage of NK cells expressing **(A)** IFNγ and **(B)** TNFα is shown. Results are presented as mean ± SE; Student’s t-test. * p < 0.05; ** p < 0.01. Each dot represents one mouse (control n=4-5/group, NK activated n=7),

**Supplemental Figure 8**. **Injection of IFNγ and TNFα reduces fibrosis MDX mice**. MDX male mice, 5 months old, were tail-vein injected with either control serum or serum supplemented with IFN-γ (50 ng/ml) and TNF-α (50 ng/ml). The transcription levels of the indicated fibrosis hallmark gene markers were analyzed in (A) the hearts, (B) the diaphragm, and (C) the skeletal muscles, measured using qRT-PCR and normalized to mb2m. Data are presented as the relative expression compared to MDX mice (determined as 1). **(D)** The heart section was stained with Masson trichrome and quantified. Each section was fully scanned. The percentage of interstitial fibrosis was determined as the ratio of the fibrosis area to the total area of the heart/tumor section using Image Pro Plus software. Data are presented as mean ± SE. One-way repeated measures ANOVA followed by Student’s t-test. **p<0.05, ** p < 0.01, ***p<0. 001. p>0.05,* no statistical significance (ns). Each dot represents one mouse (n=6-8/group).

**Supplemental Figure 9**. Gating strategy using flow cytometry in the hearts of control MDX mice (n=4) compared with IFNγ+TNFα injected mice (n=4).

**Supplemental Figure 10. IFNγ and TNFα administration reduce inflammatory gene expression in the hearts and spleens of MDX mice at both early and late time points**. MDX male mice (5 months old, n=4/group) were tail vein injected with 100µL each with either a serum derived from cancer-free MDX mice (control) or serum supplemented with IFNγ and TNFα. Mice were sacrificed as indicated. The transcription levels of the indicated inflammation hallmark gene markers were analyzed in the **(A)** hearts 24 hours post-injection, (B) spleens 24 hours post-injection, and **(C)** spleens 14 days post. Data are presented as the relative expression compared to MDX mice (determined as 1). The transcription levels of inflammation hallmark gene markers in the spleen and heart were measured using qRT-PCR, normalized to β-actin and Mb2M, respectively. Results are presented as mean ± SEM; one-way repeated measures ANOVA followed by Tukey posttest. * *p* < 0.05; ** *p* < 0.01; *** *p* < 0.001. Each dot represents one mouse.

**Supplemental Figure 11. IFN-γ and TNF-α treatment modulates TNFR1/TNFR2 signaling and reduces inflammatory gene expression in the hearts of MDX mice.** Relative mRNA levels of TNFR1 (Tnfrsf1a), TNFR2 (Tnfrsf1b), TNFα, IFNγ, IL-6, ATF3, CD163, and IL-1β were measured by qPCR in hearts of MDX mice treated with IFNγ and TNFα compared with vehicle-treated controls. Treatment decreased TNFR1 and several inflammatory markers (TNFα, CD163, IL-1β). Data are mean ± SEM. One-way repeated measures ANOVA followed by Tukey posttest; *p < 0.05, **p < 0.01, ***p<0.001, ns = not significant. Each dot represents one mouse (n=4-6/group).
