# Supplementary material for: Cancer-driven cytokine immunomodulation ameliorates cardiac function and suppresses fibrosis
Source: J Mol Cell Cardiol Plus. 2025 Nov 12;14:100493. doi: 10.1016/j.jmccpl.2025.100493 (PMC12664467; doi:10.1016/j.jmccpl.2025.100493)
Supplement: Supplementary file 2 — Supplementary tables [file mmc2.docx]

**Supplementary Table 1. The sequences of the oligonucleotides used for qRT-PCR for the indicated genes**

| **Gene** | **Forward Primer** | **Reverse Primer** |
| --- | --- | --- |
| mb2M | TTCTGGTGCTTGTCTCACTGA | CAGTATGTTCGGCTTCCCATTC |
| ACTA2 | GTCCCAGACATCAGGGAGTAA | TCGGATACTTCAGCGTCAGGA |
| Col1α1 | CTGGCGGTTCAGGTCCAAT | TTCCAGGCAATCCACGAGC |
| TGFβ3 | CCTGGCCCTGCTGAACTTG | GACGTGGGTCATCACCGAT |
| CTgF | AGACCTGTGGGATGGGCAT | GCTTGGCGATTTTAGGTGTCC |
| TIMP | GCAACTCGGACCTGGTCATAA | CGGCCCGTGATGAGAAACT |
| α-SMA | GTCCCAGACATCAGGGAGTAA | TCGGATACTTCAGCGTCAGGA |
| FN | TGGTGGCCACTAAATACGAA | GGAGGGCTAACATTCTCCAG |
| CD206 | CTAACTGGGGTGCTGACGAG | GGCAGTTGAGGAGGTTCAGT |
| Arg1 | AATGAAGAGCTGGCTGGTGT | CTGGTTGTCAGGGGAGTGTT |
| CD163 | CCTCCTCATTGTCTTCCTCCTGTG | CATCCGCCTTTGAATCCATCTCTTG |
| INFγ | ACAGCAAGGCGAAAAAGGATG | TGGTGGACCACTCGGATGA |
| TNF-α | CCCTCACACTCAGATCATCTTCT | GCTACGACGTGGGCTACAG |
| CCL2 | GTGATGGAGGGGGTCAGGA | GGGATGGGACAGCCTAAACT |
| G-CSF | CGTTCCCCTGGTCAGTGTC | CCGCTGGCCTGGATCTTC |
| IL-1b | GCAACTGTTCCTGAACTCAACT | ATCTTTTGGGGTCCGTCAACT |
| IL-13 | AACGGCAGCATGGTATGGAGTG | TGGGTCCTGTAGATGGCATTGC |
| GRP18 | TGAAGCCCAAGGTCAAGGAGAAG | TTCATGAGGAAGGTGGTGAAGGC |
| IL-6 | TACCACTTCACAAGTCGGAGGC | CTGCAAGTGCATCATCGTTGTTC |
| ATF3 | GAAGATGAGAGGAAAAGGAGGCG | GCTCAGCATTCACACTCTCCAG |
| TNFR1 | GTGTGGCTGTAAGGAGAACCAG | CACACGGTGTTCTGAGTCTCCT |
| TNFR2 | TGACAGGAAGGCTCAGATGTGC | TGACAGGAAGGCTCAGATGTGC |
| TGFB1 | TGATACGCCTGAGTGGCTGTCT | CACAAGAGCAGTGAGCGCTGAA |
| RELA | TCCTGTTCGAGTCTCCATGCAG | GGTCTCATAGGTCCTTTTGCGC |
| NFKB1 | GCTGCCAAAGAAGGACACGACA | GGCAGGCTATTGCTCATCACAG |
| Col3a1 | GACCAAAAGGTGATGCTGGACAG | CAAGACCTCGTGCTCCAGTTAG |
| MMP-9 | GCTGACTACGATAAGGACGGCA | TAGTGGTGCAGGCAGAGTAGGA |
| ERbb2 | GACCTCAGTGTCTTCCAGAACC | TGCGGTGAATGAGAGCCAATCC |
| BIRC2 | GATACGGATGAAGGGTCAGGAG | GGGTCAGCATTTTCTTCTCCTGG |

**Supplemental Table 2. Echocardiography parameters of Sup Fig.1B**

|  | **B10** | **Conrtrol** | **8 days Post inj** | **30 days  Post inj** |
| --- | --- | --- | --- | --- |
| **IVS;d-D** | 0.65±0.05 | 0.96±0.08 | 0.79±0.04 | 0.99±0.05 |
| **IVS;s - D** | 0.96±0.08 | 1.26±0.11 | 1.16±0.07 | 1.48±0.09 |
| **LVID;d - D** | 3.91±0.05 | 3.95±0.05 | 3.83±0.09 | 3.89±0.12 |
| **LVID;s - D** | 0.74±0.08 | 0.76±0.07 | 0.74±0.00 | 0.91±0.12 |
| **LVPW;d - D** | 0.74±0.04 | 0.76±0.04 | 0.74±0.03 | 0.91±0.09 |
| **LVPW;s - D** | 1.00±0.06 | 0.9±0.06 | 0.97±0.04 | 1.09±0.10 |
| **EF** | 49.75±0.83 | 40.53±1.88 | 48.94±1.60 | 52.47±1.87 |
| **FS** | 24.81±0.49 | 19.49±1.04 | 24.30±0.94 | 26.53±1.17 |

**Supplemental Table 3. Echocardiography parameters of Fig.1A**

| Days post inj | **0** | **10** | **15** | **25** |
| --- | --- | --- | --- | --- |
| **IVS;d-D** | 1.16±0.05 | 1.01±0.05 | 1.15±0.09 | 1.22±0.08 |
| **IVS;s - D** | 1.57±0.06 | 1.40±0.06 | 1.60±0.09 | 1.72±0.07 |
| **LVID;d - D** | 3.79±0.11 | 3.91±0.08 | 3.43±0.08 | 3.57±0.18 |
| **LVID;s - D** | 0.94±0.11 | 0.79±0.08 | 0.98±0.09 | 0.99±0.16 |
| **LVPW;d - D** | 0.94±0.07 | 0.79±0.04 | 0.98±0.08 | 0.99±0.08 |
| **LVPW;s - D** | 0.99±0.12 | 1.00±0.04 | 1.20±0.11 | 1.14±0.08 |
| **EF** | 44.75±1.25 | 50.42±1.99 | 56.21±1.63 | 57.95±2.34 |
| **FS** | 21.78±0.67 | 25.28±1.24 | 28.53±0.97 | 29.93±1.53 |

**Supplemental Table 4. Echocardiography parameters of Fig.1B**

| Months post inj | **3** | | **5** | | **6.5** | | **7.5** | |
| --- | --- | --- | --- | --- | --- | --- | --- | --- |
|  | **Control BS** | **Cancer BS** | **Control BS** | **Cancer BS** | **Control BS** | **Cancer BS** | **Pre inj** | **Post inj** |
| **IVS;d-D** | 0.96±0.05 | 0.96±0.05 | 0.96±0.07 | 1.11±0.07 | 0.83±0.03 | 1.00±0.11 | 0.84±0.04 | 1.08±0.07 |
| **IVS;s - D** | 0.78±0.02 | 0.82±0.04 | 1.00±0.08 | 0.81±0.04 | 1.13±0.04 | 1.38±0.15 | 1.17±0.07 | 1.29±0.16 |
| **LVID;d - D** | 2.63±0.09 | 2.64±0.09 | 3.15±0.21 | 2.63±0.11 | 3.61±0.13 | 3.62±0.08 | 3.78±0.09 | 3.46±0.33 |
| **LVID;s - D** | 1.19±0.11 | 1.12±0.11 | 0.99±0.30 | 1.37±0.12 | 0.76±0.13 | 0.79±0.07 | 0.76±0.06 | 1.03±0.32 |
| **LVPW;d - D** | 1.19±0.09 | 1.12±0.07 | 0.99±0.13 | 1.37±0.08 | 0.76±0.04 | 0.79±0.06 | 0.76±0.04 | 1.03±0.18 |
| **LVPW;s - D** | 0.83±0.08 | 0.74±0.05 | 0.90±0.11 | 0.98±0.03 | 0.90±0.04 | 1.07±0.08 | 0.97±0.07 | 1.14±0.05 |
| **EF** | 50.89±1.32 | 50.83±1.36 | 49.28±1.47 | 58.22±1.13 | 48.85±1.71 | 60.22±1.48 | 48.92±1.76 | 59.53±1.60 |
| **FS** | 25.29±0.81 | 25.26±0.83 | 24.37±0.88 | 30.15±0.7 | 24.10±0.97 | 31.44±1.03 | 24.25±1.08 | 31.07±1.07 |

**Supplemental Table 5. Echocardiography parameters Figure 2A**

|  | **naïve** | **Control BS** | **Cancer BS** |
| --- | --- | --- | --- |
| **IVS;d-D** | 0.775±0.04 | 0.801±0.08 | 0.816±0.11 |
| **IVS;s - D** | 1.195±0.07 | 1.106±0.1 | 1.23±0.17 |
| **LVID;d - D** | 3.74±0.19 | 3.866±0.46 | 3.61±0.26 |
| **LVID;s - D** | 2.531±0.12 | 3.012±0.39 | 2.64±0.16 |
| **LVPW;d - D** | 0.702±0.09 | 0.759±0.09 | 0.767±0.11 |
| **LVPW;s - D** | 1.068±0.12 | 1±0.112 | 1.07±0.064 |
| **EF** | 68.765±3.55 | 52.701±4.9 | 60.43±3.76 |
| **FS** | 32.228±2.56 | 22.162±2.58 | 26.707±2.39 |

**Supplemental Table 6. Echocardiography parameters Supplemental Figure 3**

| **Weeks post inj.** | **0** | | **6** | |
| --- | --- | --- | --- | --- |
|  | **Control BS** | **Cancer BS** | **Control BS** | **Cancer BS** |
| **IVS;d-D** | 1.18±0.079 | 0.824±0.079 | 0.935±0.065 | 0.829±0.096 |
| **IVS;s - D** | 0.88±0.04 | 1.173±0.087 | 1.267±0.043 | 1.233±0.088 |
| **LVID;d - D** | 3.82±0.27 | 3.934±0.092 | 3.819±0.157 | 3.746±0.260 |
| **LVID;s - D** | 2.91±0.2 | 2.929±0.054 | 2.895±0.099 | 2.577±0.234 |
| **LVPW;d - D** | 1.01±0.29 | 0.793±0.138 | 0.863±0.092 | 0.692±0.092 |
| **LVPW;s - D** | 1.119±0.07 | 1.182±0.200 | 1.244±0.115 | 1.258±0.458 |
| **EF** | 55.86±2.23 | 58.625±2.92 | 56.949±2.04 | 67.503±2.02 |
| **FS** | 23.87±1.28 | 25.513±1.78 | 24.209±1.13 | 31.272±1.45 |

**Supplemental Table 7. Echocardiography parameters of Sup Fig.4A**

|  | **Cancer free** | **Pre inj** | **Post inj** |
| --- | --- | --- | --- |
| **IVS;d-D** | 0.93±0.14 | 1.03±0.09 | 0.94±0.04 |
| **IVS;s - D** | 1.35±0.12 | 1.35±0.19 | 1.32±0.06 |
| **LVID;d - D** | 3.23±0.24 | 3.13±0.15 | 3.72±0.06 |
| **LVID;s - D** | 2.46±0.19 | 2.78±0.34 | 2.64±0.05 |
| **LVPW;d - D** | 1.02±0.14 | 1.13±0.10 | 0.87±0.05 |
| **LVPW;s - D** | 0.98±0.04 | 0.97±0.19 | 1.03±0.09 |
| **EF** | 48.99±1.49 | 44.673±1.03 | 56.55±124 |
| **FS** | 23.94±0.83 | 21.46±0.48 | 29.03±0.84 |

**Supplemental Table 8. Echocardiography parameters of Sup Fig.4B**

|  | **Cancer free** | **Cancer** | **Cancer heat inactivated** |
| --- | --- | --- | --- |
| **IVS;d-D** | 1.17±0.04 | 1.14±0.06 | 1.01±0.02 |
| **IVS;s - D** | 1.56±0.06 | 1.62±0.08 | 1.36±0.06 |
| **LVID;d - D** | 3.69±0.14 | 4±0.04 | 3.64±0.06 |
| **LVID;s - D** | 2.88±0.12 | 2.90±0.06 | 2.79±0.10 |
| **LVPW;d - D** | 1.01±0.05 | 0.82±0.04 | 0.89±0.05 |
| **LVPW;s - D** | 1.07±0.12 | 1.07±0.06 | 0.85±0.07 |
| **EF** | 45.25±1.34 | 54±1.95 | 47.94±2.65 |
| **FS** | 22.46±0.74 | 27.757±1.28 | 23.62±1.52 |

**Supplemental Table 9. Echocardiography parameters of Sup Fig.4C**

|  | **Pre inj** | **Post inj** |
| --- | --- | --- |
| **IVS;d-D** | 0.9±0.08 | 0.76±0.02 |
| **IVS;s - D** | 1.29±0.05 | 1.10±0.01 |
| **LVID;d - D** | 3.56±0.23 | 4.13±0.12 |
| **LVID;s - D** | 2.74±0.17 | 3.18±0.09 |
| **LVPW;d - D** | 1.11±0.13 | 0.79±0.02 |
| **LVPW;s - D** | 1.08±0.25 | 1.01±0.01 |
| **EF** | 47.40±0.85 | 46.35±1.10 |
| **FS** | 23.19.40±0.57 | 22.87.40±0.66 |

**Supplemental Table 10. Echocardiography parameters of Sup Fig.4D**

|  | **Pre inj** | **2 days Post inj** | **6 days Post inj** | **Pre inj.** | **30 days Post inj** |
| --- | --- | --- | --- | --- | --- |
| **IVS;d-D** | 1.02±0.06 | 1.01±0.12 | 0.81±0.03 | 0.89±0.10 | 0.85±0.09 |
| **IVS;s - D** | 1.32±0.07 | 1.32±0.17 | 1.14±0.11 | 1.31±0.08 | 1.17±0.10 |
| **LVID;d - D** | 3.53±0.16 | 3.54±0.22 | 3.92±0.08 | 3.90±0.15 | 3.84±0.10 |
| **LVID;s - D** | 0.96±0.13 | 2.76±0.20 | 2.74±0.09 | 2.91±0.06 | 2.92±0.09 |
| **LVPW;d - D** | 0.96±0.08 | 0.91±0.04 | 0.87±0.10 | 0.87±0.08 | 1.02±0.15 |
| **LVPW;s - D** | 1.11±0.06 | 1.00±0.07 | 1.13±0.09 | 1.06±0.08 | 1.14±0.11 |
| **EF** | 44.72±2.03 | 45±1.75 | 57.99±1.74 | 50.52±1.75 | 48.29±1.92 |
| **FS** | 21.67±1.19 | 22.9±0.90 | 30.11±1.16 | 25.30±1.17 | 23.91±1.15 |

**Supplemental Table 11. Echocardiography parameters of Fig.3A**

|  | **Pre inj** | **14 days Post inj** | **21 days Post inj** |
| --- | --- | --- | --- |
| **IVS;d-D** | 0.89±0.05 | 0.80±0.06 | 0.87±0.08 |
| **IVS;s - D** | 1.18±0.02 | 1.08±0.10 | 1.17±0.10 |
| **LVID;d - D** | 3.62±0.14 | 3.71±0.10 | 3.80±0.16 |
| **LVID;s - D** | 0.88±0.10 | 0.82±0.10 | 0.86±0.14 |
| **LVPW;d - D** | 0.88±0.04 | 0.82±0.06 | 0.86±0.07 |
| **LVPW;s - D** | 1.00±0.05 | 0.9±0.06 | 1.01±0.12 |
| **EF** | 48.73±0.93 | 49.00±1.60 | 46.51±1.27 |
| **FS** | 24.03±0.58 | 24.26±0.95 | 22.81±0.71 |

**Supplemental Table 12. Echocardiography parameters of Fig.3B**

|  | **Pre inj** | **14 days Post inj** |
| --- | --- | --- |
| **IVS;d-D** | 1.07±0.08 | 1.08±0.11 |
| **IVS;s - D** | 1.38±0.10 | 1.47±0.14 |
| **LVID;d - D** | 3.42±0.08 | 3.46±0.10 |
| **LVID;s - D** | 0.92±0.06 | 0.99±0.09 |
| **LVPW;d - D** | 0.92±0.03 | 0.99±0.08 |
| **LVPW;s - D** | 1.01±0.05 | 1.18±0.07 |
| **EF** | 49.88±0.72 | 57.53±2.42 |
| **FS** | 24.60±0.44 | 29.65±1.65 |

**Supplemental Table 13. Echocardiography parameters of Fig.4B**

| Neutralizing antibodies | **IgG** | | **G-CSF, IL-6, IFNγ, TNFα** | | **G-CSF, IL-6** | |
| --- | --- | --- | --- | --- | --- | --- |
|  | **Pre inj** | **Post inj** | **Pre inj** | **Post inj** | **Pre inj** | **Post inj** |
| **IVS;d-D** | 0.96±0.06 | 1.11±0.04 | 0.8±0.02 | 0.75±0.02 | 0.87±0.08 | 0.87±0.07 |
| **IVS;s - D** | 131±0.06 | 1.54±0.06 | 1.20±0.08 | 1.05±0.06 | 1.20±0.10 | 1.26±0.11 |
| **LVID;d - D** | 3.92±0.14 | 3.74±0.11 | 3.79±0.11 | 3.58±0.15 | 3.73±0.15 | 3.49±0.15 |
| **LVID;s - D** | 0.82±0.15 | 0.95±0.12 | 0.81±0.09 | 0.82±0.15 | 0.88±0.09 | 0.92±0.08 |
| **LVPW;d - D** | 0.82±0.04 | 0.95±0.02 | 0.81±0.04 | 0.82±0.04 | 0.88±0.06 | 0.92±0.07 |
| **LVPW;s - D** | 1.07±0.04 | 1.23±0.04 | 0.93±0.10 | 0.97±0.06 | 1.03±0.11 | 1.11±0.14 |
| **EF** | 47.44±1.94 | 55.59±2.19 | 49.90±1.89 | 53.02±2.07 | 46.58±1.58 | 58.94±1.52 |
| **FS** | 23.44±1.09 | 28.37±1.40 | 24.87±1.18 | 26.67±1.19 | 22.69±1.05 | 30.47±1.10 |

| Neutralizing antibodies | **IFNγ, TNFα** | | **IFNγ** | | **TNFα** | |
| --- | --- | --- | --- | --- | --- | --- |
|  | **Pre inj** | **Post inj** | **Pre inj** | **Post inj** | **Pre inj** | **Post inj** |
| **IVS;d-D** | 0.77±0.04 | 0.78±0.06 | 0.73±0.02 | 0.79±0.02 | 0.90±0.05 | 0.82±0.04 |
| **IVS;s - D** | 1.11±0.02 | 1.17±0.08 | 1.02±0.03 | 1.12±0.02 | 1.15±0.14 | 1.05±0.11 |
| **LVID;d - D** | 3.73±0.22 | 3.46±0.11 | 3.71±0.24 | 3.66±0.26 | 3.53±0.37 | 3.29±0.29 |
| **LVID;s - D** | 0.83±0.17 | 0.82±0.11 | 0.75±0.19 | 0.85±0.24 | 0.91±0.11 | 0.88±0.20 |
| **LVPW;d - D** | 0.83±0.06 | 0.82±0.03 | 0.75±0.05 | 0.85±0.02 | 0.91±0.10 | 0.88±0.11 |
| **LVPW;s - D** | 0.98±0.06 | 0.99±0.04 | 0.94±0.07 | 1.01±0.04 | 0.87±0.05 | 0.92±0.06 |
| **EF** | 48.42±0.47 | 53.45±3.25 | 48.49±1.24 | 52.59±3.15 | 48.42±3.98 | 53.73±1.87 |
| **FS** | 23.88±0.22 | 26.95±2.07 | 23.92±0.68 | 26.49±1.86 | 24.07±2.36 | 27.10±1.17 |

**Supplemental Table 14. Echocardiography parameters of Fig.4C**

|  | **Pre inj** | **10 days Post inj** |
| --- | --- | --- |
| **IVS;d-D** | 1.04±0.06 | 1.28±0.03 |
| **IVS;s - D** | 0.85±0.04 | 0.97±0.07 |
| **LVID;d - D** | 2.74±0.09 | 2.49±0.07 |
| **LVID;s - D** | 1.12±0.13 | 1.64±0.05 |
| **LVPW;d - D** | 1.12±0.06 | 1.64±0.11 |
| **LVPW;s - D** | 0.84±0.07 | 1.27±0.04 |
| **EF** | 50.90±0.67 | 54.62±1.91 |
| **FS** | 25.37±0.45 | 27.61±1.24 |

**Supplemental Table 15. Echocardiography parameters of Sup Fig.5A**

|  | **Control BS** | | | **Control BS- IFNγ+TNFα** | | |
| --- | --- | --- | --- | --- | --- | --- |
|  | **Pre inj** | **7 days Post inj** | **14 days Post inj** | **Pre inj** | **7 days Post inj** | **14 days Post inj** |
| **IVS;d-D** | 0.81±0.07 | 0.91±0.12 | 0.82±0.08 | 0.99±0.04 | 0.95±0.06 | 0.95±0.05 |
| **IVS;s - D** | 1.13±0.1 | 1.28±0.13 | 1.19±0.07 | 1.32±0.04 | 1.25±0.09 | 1.34±0.06 |
| **LVID;d - D** | 3.86±0.13 | 3.87±0.16 | 3.89±0.13 | 3.85±0.12 | 3.49±0.11 | 3.47±0.17 |
| **LVID;s - D** | 0.87±0.10 | 0.18±0.10 | 0.82±0.09 | 0.92±0.12 | 0.79±0.11 | 0.94±0.14 |
| **LVPW;d - D** | 0.87±0.05 | 0.81±0.06 | 0.82±0.07 | 0.92±0.06 | 0.79±0.03 | 0.94±0.04 |
| **LVPW;s - D** | 0.98±0.06 | 0.88±0.08 | 0.95±0.04 | 1.01±0.07 | 1.01±0.03 | 1.19±0.06 |
| **EF** | 47.49±0.71 | 44.78±1.07 | 46.27±0.33 | 45.67±2.72 | 57.22±1.11 | 60.79±2.32 |
| **FS** | 23.41±0.44 | 21.83±0.69 | 22.50±0.38 | 22.44.±1.61 | 29.31±0.71 | 31.80±1.51 |

**Supplemental Table 16. Echocardiography parameters of Sup Fig.5D**

|  | **CSF1R depleted** | | **No depletion IFNγ+TNFα** | | **CSF1R depleted+ IFNγ+TNFα** | |
| --- | --- | --- | --- | --- | --- | --- |
|  | **Pre inj** | **Post inj** | **Pre inj** | **Post inj** | **Pre inj** | **Post inj** |
| **IVS;d-D** | 0.99±0.12 | 1.03±0.10 | 0.97±0.08 | 0.93±0.09 | 0.95±0.06 | 0.94±0.06 |
| **IVS;s - D** | 1.35±0.16 | 1.37±0.10 | 1.34±0.08 | 1.32±0.12 | 1.23±0.09 | 1.25±0.09 |
| **LVID;d - D** | 3.56±0.23 | 3.52±0.19 | 3.78±0.07 | 3.36±0.05 | 3.43±0.09 | 3.43±0.14 |
| **LVID;s - D** | 0.98±0.19 | 1.08±0.15 | 0.94±0.10 | 0.88±0.06 | 0.90±0.07 | 0.97±0.10 |
| **LVPW;d - D** | 0.98±0.13 | 1.08±0.08 | 0.94±0.06 | 0.88±0.03 | 0.90±0.03 | 0.97±0.06 |
| **LVPW;s - D** | 0.94±0.15 | 1.10±0.05 | 1.05±0.03 | 0.99±0.05 | 1.03±0.01 | 1.03±0.04 |
| **EF** | 46.99±1.29 | 47.72±0.65 | 44.95±2.73 | 57.15±1.50 | 48.37±0.79 | 48.96±1.41 |
| **FS** | 22.49±0.68 | 23.35±0.33 | 21.93±1.54 | 29.15.±0.95 | 23.70±0.47 | 24.09±0.89 |

**Supplemental Table 17. Echocardiography parameters Figure 6A**

| **weeks post inj.** | **0** | | **1** | | **2** | |
| --- | --- | --- | --- | --- | --- | --- |
|  | **Control** | **IFN𝛾+TNF𝛼** | **Control** | **IFN𝛾+TNF𝛼** | **Control** | **IFN𝛾+TNF𝛼** |
| **IVS;d** | 0.848±0.11 | 0.774±0.03 | 0.829±0.08 | 0.818±0.1 | 0.856±0.08 | 0.843±0.08 |
| **IVS;s** | 1.198±0.12 | 1.053 ± 0.03 | 1.146±0.14 | 1.209±0.18 | 1.176±0.1 | 1.226±0.1 |
| **LVID;d** | 3.549±0.23 | 3.881±0.4 | 3.730±0.19 | 3.842±0.16 | 3.671±0.1 | 4.01±0.29 |
| **LVID;s** | 2.626±0.13 | 2.972±0.3 | 2.861±0.23 | 2.73±0.12 | 2.853±0.08 | 2.691±0.38 |
| **LVPW;d** | 0.761±0.07 | 0.762±0.09 | 0.769±0.14 | 0.781±0.1 | 0.805±0.07 | 0.784±0.1 |
| **LVPW;s** | 1± 0.07 | 1.044±0.1 | 1.060±0.16 | 1.07±0.13 | 1.056±0.09 | 1.169±0.14 |
| **EF** | 51.884±3.16 | 47.250±5.02 | 47.681±5.02 | 56.74±5.2 | 45.716±2.3 | 61.5±7.62 |
| **FS** | 25.933±2.27 | 23.52±2.9 | 23.202±3.01 | 28.88±3.74 | 22.560±1.2 | 33.09±5.8 |
